# A Nanoradiomics Approach for Differentiation of Tumors Based on Tumor-Associated Macrophage Burden

**DOI:** 10.1155/2021/6641384

**Published:** 2021-06-14

**Authors:** Zbigniew Starosolski, Amy N. Courtney, Mayank Srivastava, Linjie Guo, Igor Stupin, Leonid S. Metelitsa, Ananth Annapragada, Ketan B. Ghaghada

**Affiliations:** ^1^Edward B. Singleton Department of Radiology, Texas Children's Hospital, Houston, TX, USA; ^2^Department of Radiology, Baylor College of Medicine, Houston, TX, USA; ^3^Texas Children's Cancer Center, Department of Pediatrics, Baylor College of Medicine, Houston, TX, USA; ^4^Center for Cell and Gene Therapy, Baylor College of Medicine, Houston, TX, USA

## Abstract

**Objective:**

Tumor-associated macrophages (TAMs) within the tumor immune microenvironment (TiME) of solid tumors play an important role in treatment resistance and disease recurrence. The purpose of this study was to investigate if nanoradiomics (radiomic analysis of nanoparticle contrast-enhanced images) can differentiate tumors based on TAM burden.

**Materials and Methods:**

In vivo studies were performed in transgenic mouse models of neuroblastoma with low (*N* = 11) and high (*N* = 10) tumor-associated macrophage (TAM) burden. Animals underwent delayed nanoparticle contrast-enhanced CT (n-CECT) imaging at 4 days after intravenous administration of liposomal-iodine agent (1.1 g/kg). CT imaging-derived conventional tumor metrics (tumor volume and CT attenuation) were computed for segmented tumor CT datasets. Nanoradiomic analysis was performed using a PyRadiomics workflow implemented in the quantitative image feature pipeline (QIFP) server containing 900 radiomic features (RFs). RF selection was performed under supervised machine learning using a nonparametric neighborhood component method. A 5-fold validation was performed using a set of linear and nonlinear classifiers for group separation. Statistical analysis was performed using the Kruskal–Wallis test.

**Results:**

N-CECT imaging demonstrated heterogeneous patterns of signal enhancement in low and high TAM tumors. CT imaging-derived conventional tumor metrics showed no significant differences (*p* > 0.05) in tumor volume between low and high TAM tumors. Tumor CT attenuation was not significantly different (*p* > 0.05) between low and high TAM tumors. Machine learning-augmented nanoradiomic analysis revealed two RFs that differentiated (*p* < 0.002) low TAM and high TAM tumors. The RFs were used to build a linear classifier that demonstrated very high accuracy and further confirmed by 5-fold cross-validation.

**Conclusions:**

Imaging-derived conventional tumor metrics were unable to differentiate tumors with varying TAM burden; however, nanoradiomic analysis revealed texture differences and enabled differentiation of low and high TAM tumors.

## 1. Introduction

The immunosuppressive tumor microenvironment (TME) is a major contributor of treatment resistance and disease recurrence in solid tumor malignancies [1, 2]. Among various immune cell types, tumor-associated macrophages (TAMs) represent a key cellular player and therapeutic target [3–6]. Growing evidence suggests that TAMs carry out critical roles in essentially every stage of disease progression including tumor growth, angiogenesis, metastasis, and treatment resistance to conventional and emerging targeted therapies [7, 8]. A high burden of TAMs has been correlated with metastatic phenotype, treatment resistance, and poor prognosis in adult and pediatric solid tumors [9–13]. Techniques for monitoring TAM burden in solid tumors could aid in disease prognosis, a priori identification of treatment resistance, and monitoring of tumor response to TAM-directed immunotherapies.

Biopsy-based approaches are challenging for accurate assessment and monitoring of TAM burden due to spatial tumor heterogeneity and difficulties in repeated invasive tumor sampling procedures. Conventional imaging methods and standard imaging quantitative metrics based on ultrasound, contrast-enhanced CT, and MRI are insensitive to TAM burden since macrophages represent a relatively small fraction (<10%) of cells within the tumor mass and exert an indirect effect on tumor growth. Molecular imaging using TAM-targeted contrast agents is undergoing investigation for use with MRI and PET imaging [14–17].

Due to the central role of TAMs in angiogenesis and development of tumor vascular architecture, advanced algorithms that quantitatively analyze images to search for subtle changes in tumor morphology and texture could help in differentiating tumors with varying TAM levels. Radiomics is a growing field in diagnostic radiology, wherein in vivo images are mined to objectively define phenotypic characteristics of tumors by extracting quantitative image features such as intensity, shape, size, morphology, and texture [18, 19]. In this study, we performed radiomic analysis on contrast-enhanced CT (CECT) images acquired using a nanoparticle contrast agent that enables interrogation of tumor vascular architecture [20–22]. We investigated if nanoradiomics (radiomic analysis of nanoparticle contrast-enhanced images) can differentiate tumors based on TAM burden.

## 2. Materials and Methods

### 2.1. Animals Models with Varying TAM Burden

In vivo studies were performed in transgenic mouse models of neuroblastoma. Transgenic mice that develop spontaneous bilateral adrenal neuroblastoma tumors driven by tetracycline inducible simian virus 40 T-antigen (SV40-Tag or NB-Tag) were a gift from Dr. H. Iwakura (Kyoto University, Japan) and used as a model of a relatively low TAM burden [23, 24]. We crossed NB-Tag with CD1d^−/−^ mice (a gift from Dr. L. Van Kaer, Vanderbilt University, Nashville, TN) which are deficient in natural killer T (NKT) cells [25] to generate NB-Tag/CD1d^−/−^ mice. Since NKT cells control TAMs in neuroblastoma [26, 27], NB-Tag/CD1d^−/−^ mice were used as a model of high TAM burden. All mice were on a C57BL/6 background. A total of 11 mice were included in the low TAM group and 10 mice were included in the high TAM group. Out of these, 6 mice/group were used for the determination of TAM burden by flow cytometry. The remaining mice (*N* = 5 in the low TAM group and *N* = 4 in the high TAM group) were used for the CT imaging study. Since transgenic mice develop bilateral tumors, *n* = 10 low TAM tumors and *n* = 8 high TAM tumors were included in the radiomic analysis.

### 2.2. Nanoparticle Contrast Agent

A PEGylated liposomal-iodine nanoparticle contrast agent was used for CECT imaging [20, 21]. Phospholipids consisting of 1,2-dipalmitoyl-sn-glycero-3-phosphocholine, cholesterol, and 1,2-distearoyl-sn-glycero-3-phosphoethanolamine-N-[methoxy (polyethylene glycol)-2000] were dissolved in ethanol at a molar ratio of 56 : 40 : 4. An aqueous solution of iodixanol (550 mg I/mL) was added to ethanolic lipid solution and hydrated for 45 minutes followed by sequential extrusion at −65°C for particle sizing. The resultant solution was diafiltered against 150 mM saline/10 mM histidine solution to remove unencapsulated iodixanol. The average liposome size in the final formulation, determined by dynamic light scattering (DLS), was 128 ± 16 nm. The iodine concentration, determined by UV spectrophotometry (*λ*_245 nm_), was 105 ± 6 mg I/mL.

### 2.3. Nanoparticle CECT Imaging

Delayed nanoparticle CECT (n-CECT) imaging was performed four days after intravenous administration of nanoparticle contrast agent (1.1 g I/kg) [21]. Imaging was performed on a small animal micro-CT system (Inveon, Siemens Inc., Knoxville, TN, USA). Mice were anesthetized using −3% isoflurane, positioned on a CT scanner bed and maintained at −1.5% isoflurane delivered via nose cone. The following scan parameters were used for the acquisition of CT images: 70 kVp, 0.5 mA, 850 ms X-ray exposure, 540 projections, −20 min scan time. Images were reconstructed at an isotropic resolution of 35 *μ*m and calibrated for Hounsfield unit (HU).

### 2.4. Determination of TAM Burden

Tumors were harvested and processed to obtain single cell suspensions for determination of TAM burden by flow cytometry. Briefly, minced tumor tissues were digested with collagenase IV, dispase II, and DNAse I (Sigma). Single cell suspension was then treated with ACK lysing buffer (Lonza) to remove red blood cells, and immune cells were isolated using a Percoll (GE Healthcare) gradient [28]. Cells were stained with CD45 PE, CD11b PerCP, F4/80 Pacific Blue, Ly6C FITC, Ly6G APC-Cy7 (BD Biosciences), and the Aqua Live/Dead viability dye (Molecular Probes). Cells were then analyzed on the LSRII four-laser flow cytometer (BD Biosciences) using BD FACSDiva software (version 6.0) and FlowJo (version 7.2.5; BD Biosciences). TAMs were defined as percentage of CD45 cells that were CD11b^+^, Ly6G^−^, Ly6C^−^, and F4/80^+^ [29].

### 2.5. Immunofluorescence Analysis

Frozen tumor tissue was sectioned (5 *μ*m) and transferred to slides. Sections were washed with phosphate-buffered saline (PBS) for 5 minutes to remove optimal cutting temperature (OCT) medium, and the tissue was fixed with 4% formalin for 15 minutes. The sections were again washed three times with PBS for 5 minutes each. Sections were permeabilized using 0.01% Triton in PBS for 15 minutes. At the end of incubation, sections were washed 3 times with PBS. Nonspecific sites on tissue sections were blocked using 5% BSA in PBS. Subsequently, sections were incubated with anti-CD11b-AF488 (1 : 50 dilution) and anti-CD31 (1 : 100 dilution) overnight at 4C. The sections were washed three times with PBST (PBS + 0.01% Tween 20) for 5 minutes each. Sections were then incubated with anti-AF647 (appropriate secondary for CD31) for 90 minutes at room temperature. Sections were again washed three times with PBST for 5 minutes each and incubated with DAPI (1 *μ*g/ml in PBS) for 5 minutes. The sections were mounted using antifade mounting media and imaged on an Olympus Fluoview confocal microscope (FV10000) for CD11b (AF-488, Ex/Em: 488/525 nm), CD31 (AF-647, Ex/Em: 594/650 nm), and DAPI nuclei (Ex/Em: 405/470 nm) staining.

Five fields of interest (60x magnification) were imaged from each section for quantitative analysis. 5-6 tumor sections were analyzed from each tumor specimen. CD11b^+^ spots were manually counted around CD31^+^ areas. Analysis of perivascular CD11b^+^ TAMs was performed by counting CD11b^+^ cells within 3 cell layers (identified by DAPI stained nucleus) of CD31^+^ cells. Remaining CD11b^+^ cells were considered as nonperivascular macrophages [30, 31].

### 2.6. Radiomics and Image Analysis

**Figure 1:**
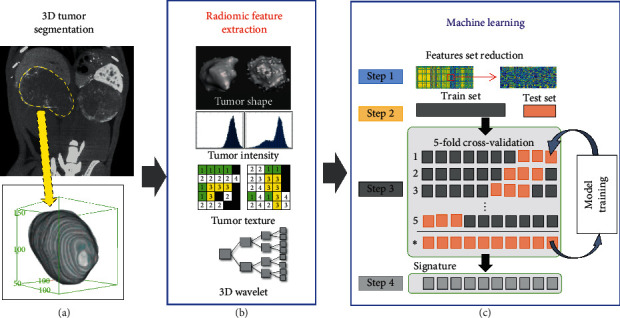
Pipeline for determination of radiomic signatures. (a) 3D tumor segmentation of n-CECT images. (b) Radiomic features extraction (900 radiomics features: shape, intensity texture based, 3D wavelets). (c) Machine learning: feature selection and reduction of highly correlated features, followed by model training and 5-fold cross-validation for identifying radiomic signature.

Radiomic analysis was combined with machine learning for determining radiomic signatures that classify tumors based on TAM burden (Figure 1). Regions of interest (ROIs) were manually drawn in delayed n-CECT images to delineate tumor margins for 3D segmentation of tumor volume. CT imaging-derived conventional tumor metrics were computed for segmented tumors. These included tumor volume and tumor mean CT attenuation. Radiomic analysis was performed using a PyRadiomics workflow implemented in the quantitative image feature pipeline (QIFP) server containing a total of 900 radiomic features (RFs) to analyze shape, size, intensity, morphology, and texture [32]. These radiomic features are divided into three categories: (1) original radiomics features (*n* = 86) which contain first-order statistics, shape and size descriptors, and texture classes which include gray-level co-occurrence matrix (GLCM), gray level run length matrix (GLRLM), gray level size zone matrix (GLSZM), gray level dependence matrix (GLDM), and neighboring gray-tone difference matrix (NGTDM); (2) logarithmic enhancement of original radiomics features with three degrees of Sigma values (*n* = 222); and (3) wavelet representations of original radiomic features (*n* = 592). A mathematical basis and detailed description for each radiomic feature has been described previously [33].

RF selection was performed using the nonparametric neighborhood component method [34]. The method uses a gradient ascent technique (a diagonal adaptation of neighborhood component analysis) to maximize the expected leave-one-out classification accuracy with a regularization threshold set to 0.2 [35]. RFs reflecting tumor size was discarded (*n* = 48) due to potential concerns of size bias. As a result, RF selection was performed on a reduced set (*n* = 852 RFs) using the nonparametric neighborhood component method followed by elimination of highly correlated features with absolute value of Pearson correlation coefficient > 0.7. The resulting vector of radiomic features was fed into a supervised machine learning module (in MATLAB) containing various classifiers for identification of radiomic signatures.

### 2.7. Statistical Analysis

Statistical analysis of flow cytometry-derived TAM burden was performed using the Wilcoxon rank sum test. CT imaging-derived conventional tumor metrics were analyzed using the Kruskal–Wallis test. A 5-fold cross-validation was performed using a set of linear and nonlinear classifiers to confirm the accuracy for group separation.

## 3. Results

**Figure 2:**
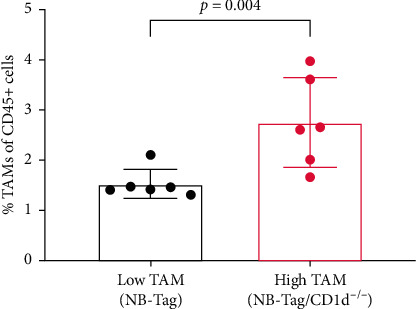
Tumor-associated macrophage (TAM) burden. The levels of TAMs were determined by flow cytometry. TAM burden is expressed as a percentage of CD45^+^ cells that are CD11b^+^/F4/80^+^/Ly6C^−^/Ly6G^−^. Data are presented as mean and standard deviation. ^*∗*^Significant difference (*p* < 0.05).

**Figure 3:**
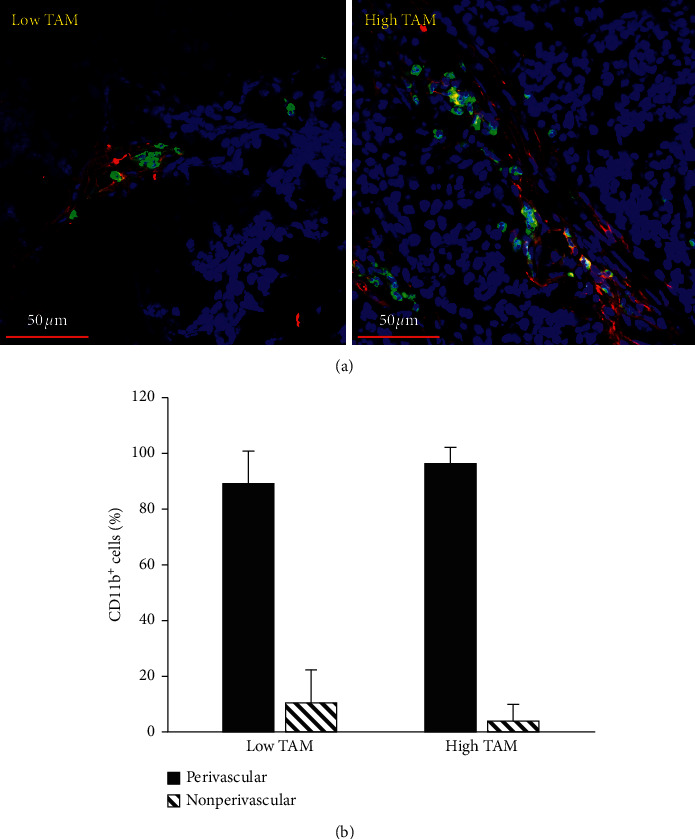
Perivascular distribution of tumor-associated macrophages (TAMs). (a) Representative immunofluorescence images demonstrate a predominant perivascular distribution of TAMs in low and high TAM burden tumors. TAMs were stained with CD11b-AF647 (green) and blood vessels were stained with CD31-AF477 (red). (b) Distribution of CD11b + TAMs in perivascular and nonperivascular regions.

Flow cytometry was performed to measure the overall burden on TAMs in tumors of NB-Tag/CD1d^−/−^ (referred as high TAM tumors) and NB-Tag mice (referred as low TAM tumors). Flow cytometry analysis demonstrated a significantly higher (*p* < 0.05) burden of CD11b^+^/F4/80^+^/Ly6C^−^/Ly6G^−^ TAMs in high TAM tumors compared to low TAM tumors (Figure 2). The spatial distribution of TAMs relative to tumor vasculature was assessed by fluorescence microscopy. Immunofluorescence analysis revealed a predominant perivascular distribution of TAMs in both tumor models (Figure 3).

**Figure 4:**
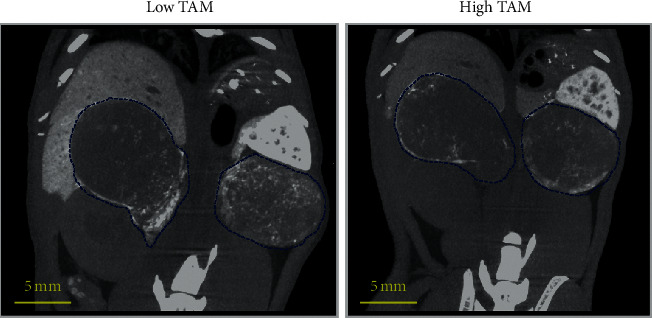
Nanoparticle contrast-enhanced CT. Representative thick slab coronal n-CECT images of mouse lower abdomen demonstrating heterogeneous pattern of signal enhancement in spontaneous bilateral tumors (blue contours) in low TAM and high TAM tumors.

**Figure 5:**
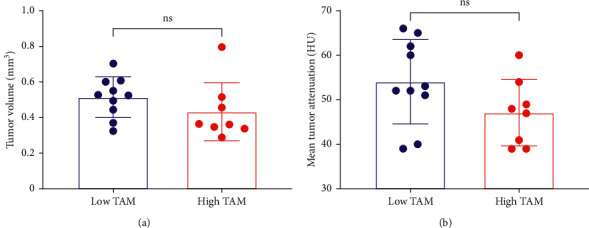
n-CECT-derived conventional tumor metrics. (a) CT-derived tumor volume and (b) mean CT attenuation of tumors in low TAM and high TAM tumors. Data are presented as mean and standard deviation. ns, not significant.

Delayed contrast-enhanced CT imaging was performed four days after administration of nanoparticle contrast agent. A four-day time point was chosen to give adequate time for the nanoparticle contrast agent to extravasate in tumor and ensure its clearance from systemic circulation, thereby eliminating potential confounding signal from residual blood-pool signal [21]. Nanoparticle contrast-enhanced CT (n-CECT) imaging demonstrated heterogeneous patterns of signal enhancement in bilateral tumors in low and high TAM tumor models (Figure 4). CT-derived conventional tumor metrics showed no significant differences (*p* > 0.05) in tumor volume between low TAM (0.51 ± 0.11 cm^3^) and high TAM (0.43 ± 0.16 cm^3^) tumors (Figure 5(a)). Tumor CT attenuation did not differ significantly (*p* > 0.05) between low TAM (54 ± 9 HU) and high TAM (47 ± 7 HU) tumors (Figure 5(b)). Overall, these findings suggest that CT-derived conventional tumors metrics are unable to differentiate tumors based on their TAM burden.

**Figure 6:**
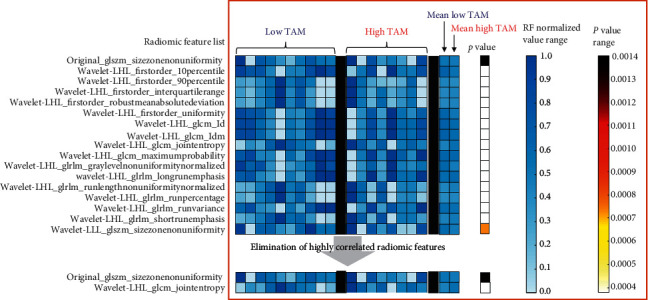
Radiomic analysis of low and high TAM tumors. List of RFs that differentiated low TAM and high TAM tumor groups. Highly correlated RFs were eliminated, resulting in two RFs, which when fed into a linear classifier yielded an accuracy of 100% for classification and differentiation of tumors based on TAM burden. Each column in low TAM and high TAM groups shows a vector of normalized RF values representing individual tumor sample. Mean values of each RF were calculated for each group. Statistical analysis was performed using the Kruskal–Wallis test to identify RFs that differentiated low TAM and high TAM groups. *p* < 0.05 was considered statistically significant.

**Figure 7:**
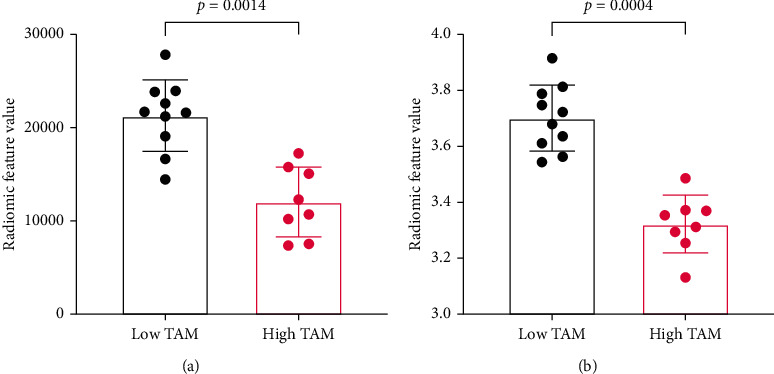
Examples of radiomic features that differentiate low TAM and high TAM tumors. (a) Original GLSZM size zone nonuniformity. (b) Wavelet LHL GLCM joint entropy.

Advanced quantitative analysis was performed on three-dimensional (3D) images for differentiating tumors based on their TAM burden. Tumors segmented in n-CECT images were processed using the open-access QIFP radiomics software for computing radiomic features (RFs). A total of 852 RFs (48 out of 900 RFs were excluded due to potential size bias) representing tumor's phenotypic characteristics were computed for each segmented tumor CT dataset. A diagonal adaptation of neighborhood component analysis was utilized to identify radiomic features that differentiated tumors based on TAM burden. A total of 17 RFs were identified (Figure 6), out of which 15 highly correlated wavelet-based RFs (absolute value of Pearson correlation coefficient > 0.7) were eliminated. The remaining two RFs were fed into a machine learning module for training linear and nonlinear classifiers. A linear classifier-based trained model yielded a perfect validation accuracy of 1.0 for the classification and differentiation of low TAM and high TAM tumors (Figure 6). A 5-fold validation of RFs was performed using a machine learning approach to confirm the high accuracy of the linear classifier (RF signature) that differentiated tumors based on TAM burden. The identified RFs belonged to two categories: original gray level size zone matrix (GLSZM) (one RF) and second category wavelet (one RF) belonging to the wavelet LHL (low-high-low) subcategory (Figure 7).

## 4. Discussion

Tumor-associated macrophages (TAMs), a key component of the TME, orchestrate several functions in mediating immune suppression and disease progression [4, 8]. Noninvasive methods that differentiate TAM burden in tumors could enable patient stratification and interventions to facilitate improved treatment outcomes. In this work, we investigated a radiomics approach for differentiating tumors based on TAM burden. This study demonstrated that while CT imaging-derived conventional tumor metrics cannot differentiate tumors with varying TAM burden, radiomic analysis of nanoparticle contrast-enhanced images can differentiate and classify tumors based on their TAM burden.

Current research on noninvasive approaches for the determination of TAM burden has primarily focused on using molecular imaging agents. Both small molecule and nanoparticle platform-based imaging agents have been investigated using MRI and nuclear techniques (PET/SPECT) as the read-out [14, 15, 17, 36]. Such approaches have the advantage of identifying specific locations in tumor with high TAM levels. However, these approaches pose a challenge in TAM-based tumor classification when TAM levels do not vary substantially or when the mean tumor signal is confounded by nonspecific signal (signal from passive intratumoral localization of TAM-targeted imaging probes). In our work, we investigated a radiomics approach to data mine images and detect subtle changes in tumor architecture and texture due to varying TAM levels.

Recent studies have revealed that TAMs predominantly exhibit a perivascular distribution in tumor, and this pattern of distribution is likely responsible for treatment resistance and metastatic progression [5]. Our work also demonstrated a predominant perivascular distribution of TAMs in the studied mouse models of spontaneous neuroblastoma tumors. Due to their perivascular distribution, TAMs play a central role in shaping tumor vasculature [5, 30, 31]. Therefore, we investigated if advanced quantitative imaging methods that interrogate whole tumor vasculature could aid in classifying tumors based on their TAM burden. Studies were performed in transgenic mice with spontaneous tumors. The use of spontaneous tumor models ensures natural tumor development in immune competent mice without external intervention to artificially manipulate the tumor architecture and tumor microenvironment. This is particularly important in the setting of radiomic analysis which seeks to discern texture-based architectural difference in tumor phenotype.

A nanoparticle contrast agent was utilized in combination with high-resolution CT imaging to probe tumor vasculature in models of varying TAM burden. Previous studies have shown utility of nanoparticle CT contrast agents in probing tumor vasculature for differentiation of tumor phenotypes and monitoring tumor response to vascular-targeted therapies [37–39]. Unlike conventional contrast agents which have rapid wash-in/wash-out kinetics in tumors, nanoparticle contrast agents extravasate and reside in tumors for prolonged period due to enhanced permeability and retention (EPR) phenomenon associated with abnormal tumor vasculature [20, 40]. As a result, the pattern of nanoparticle contrast distribution in tumors is strictly governed by tumor architecture and therefore allows interrogation of TAM influence on the pattern of tumor vascular architecture and changes in vascular permeability. The prolonged retention of nanoparticle contrast agents in tumors provides a longer window to capture contrast-enhanced images without concerns of signal variability/decay which is often observed with rapid injection of conventional agents due to their short half-life. These properties would benefit radiomic analysis of nanoparticle contrast-enhanced images since it eliminates artificial variations in signal enhancement patterns associated with rapid tumor kinetics of the contrast agent, a phenomenon observed with conventional low molecular weight imaging agents [41, 42].

Analysis of CT imaging-derived conventional tumor CT metrics did not reveal differences in tumor size or mean tumor CT attenuation between low and high TAM burden tumors. These findings are consistent with a previous study which demonstrated that subtle changes in tumor immune burden do not cause overall changes detectable with conventional imaging [22].

Radiomic analysis was performed on high-resolution CECT images. CT has been at the forefront for radiomic analysis in clinical domain due to its quantitative nature (CT attenuation is directly proportional to contrast agent concentration), high spatial resolution, and isotropic voxels, thereby facilitating reproducibility, ease of standardization for multicenter and widespread use [43, 44]. Radiomic analysis of delayed nanoparticle CECT images, however, revealed differences in tumor texture between low TAM and high TAM tumors. A 5-fold validation was implemented to improve robustness of RFs in classifying tumors. The discovered radiomic signatures consisted of two RFs, comparable to results of previous studies [45, 46]. The emergence of these RFs suggests that wavelet decomposition of nanoparticle CECT images provides multifrequency representations that uncover information about tumor heterogeneity and TAM burden. While it is difficult to pinpoint the precise molecular and cellular perturbations that result in the identification of RFs, it is acknowledged that understanding the biological basis of RFs will be important to advance the field towards clinical translation [47, 48]. The robustness and utility of nanoradiomics was shown recently in another study, wherein it allowed for monitoring the efficacy of TME-directed cellular immunotherapy by differentiating tumors with varying myeloid-derived suppressor cells (MDSCs) levels [22]. Similar to this study, the previous study also revealed texture-based nanoradiomic features in detecting MDSC-induced tumor vascular architecture changes [22].

We acknowledge that our study has limitations. The nanoradiomics approach needs to be validated in additional tumor models of varying TAM burden. The generation of spontaneous tumors with varying TAM levels in immune competent mice is not trivial. Although TAM levels can theoretically be varied using mouse models of implantable tumors wherein tumor cells are coimplanted with TAMs, such methods present challenges with precise control of TAM burden. While the tumor models utilized in this study clearly showed differences in TAM levels, it is possible that other components of TME may also be naturally affected given the highly complex interplay between TAMs, other myeloid and immune cells, and tumor cells within the TME. Radiomic analysis was performed on a small sample size (*n* = 8–10 tumors). It is possible that with larger sample size, the accuracy could be lower. We performed nanoradiomics on CECT images due to the higher spatial resolution (35 *μ*m), isotropic voxel, and quantitative nature of CT. However, further studies are warranted to understand the effect of CT scan parameters on robustness of radiomic features. We did not compare nanoradiomic features against features generated from radiomic analysis of noncontrast CT images since such analysis is unlikely to provide robust radiomic features due to lack of intratumoral signal variations in noncontrast CT images. We could not compare results of nanoradiomics against conventional radiomic analysis (analysis of CECT images acquired using a conventional contrast agent) due to the inherent limitations (long scan times) of small animal CT scanners which makes conventional CECT impractical. However, it is likely that radiomic analysis can be performed on contrast-enhanced MR images acquired using nanoparticle contrast agents, such as ultrasmall superparamagnetic iron oxide (USPIO) nanoparticles. Finally, in addition to intratumoral region, radiomic analysis of tumor periphery or peritumoral region could potentially aid in a comprehensive understanding of the tumor microenvironment.

## 5. Conclusion

This study shows that tumors with different TAM burden manifest subtle changes in tumor architecture that are not detected with imaging-derived conventional tumor metrics but are revealed on radiomic analysis of nanoparticle contrast-enhanced images.

## Data Availability

The data used to support the findings of this study are available from the corresponding author upon request.
